# Tumor necrosis factor-α-induced a disintegrin and metalloprotease 10 increases apoptosis resistance in prostate cancer cells

**DOI:** 10.3892/ol.2014.1810

**Published:** 2014-01-16

**Authors:** LI BING ZHU, SHENG TAO ZHAO, TING ZHAO XU, HE WANG

**Affiliations:** 1 Department of Urology, The People’s Liberation Army Mount Lu Sanatorium, Jiujiang, Jiangxi 332000, P.R. China; 2 Department of Urology, Fuzhou General Hospital of Nanjing Military Area Command, Fuzhou, Fujian 350000, P.R. China; 3 Department of Respiratory Medicine, Kunming General Hospital of Chengdu Military Area Command, Kunming, Yunnan 650000, P.R. China; 4 Department of Urology, Tangdu Hospital, The Fourth Military Medical University, Xi’an, Shaanxi 710038, P.R. China

**Keywords:** prostate cancer, a disintegrin and metalloproteinase 10, fas ligand, tumor necrosis factor-α, apoptosis

## Abstract

In developed countries, prostate cancer (PCa) is the second most frequently diagnosed type of cancer and the third most common cause of cancer-related mortality in males. Compared with western countries, the morbidity rate of PCa in China is markedly lower, however, it is rising annually. The etiology of PCa is unclear, therefore, to investigate how a disintegrin and metalloprotease 10 (ADAM10) functions in PCa, ADAM10 mRNA and protein levels induced by tumor necrosis factor (TNF)-α were identified using polymerase chain reaction and flow cytometry, respectively. To investigate the mechanism of ADAM10 activity in PCa, specific inhibitors were used, and DNA transfection and RNA interference technology were employed to identify the interaction between ADAM10 and the Fas ligand (L). The results indicated that TNF-α induced ADAM10 expression in a time- and dose-dependent manner through the p38 mitogen-activated protein kinase/necrosis factor-κB signaling pathway. ADAM10 hydrolyzed FasL and contributed to apoptosis resistance of the tumor cells. These observations indicate a promising therapeutic modality for the treatment of apoptosis-resistant PCa, by targeting ADAM10 sheddase activity.

## Introduction

Prostate cancer (PCa) is a complex and biologically heterogeneous disease (1) and there is currently no cure for advanced, hormone-refractory PCa (2). PCa is the second leading cause of cancer mortality in males >40 years of age in the USA (3) and the third most common cause of cancer-related mortality in males (4). PCa is generally a slow developing type of cancer, and 5- and 10-year relative survival rates of early stage PCa are 99 and 95%, respectively (5). Compared with western countries, the Chinese population exhibits a lower incidence of PCa, however, it is increasing annually, in addition to an increased average life expectancy, improved dietary patterns and enhanced diagnosis technology. There are numerous risk factors that induce PCa, such as specific hormones, age, ethnicity and family history. Regardless of other factors, a family history of PCa is the strongest known risk factor (6).

Speculation regarding an association between inflammation and cancer has been considered for some time and epidemiological studies have established that numerous tumors occur alongside chronic infectious diseases (7). Prostatitis is a common clinical disease, which is associated with renal surgery and it is also known that diet and sexually transmitted infections increase the risk of PCa (8). During the development of PCa, tumor cells and the microenvironment of the local host tissue interact and form a tumor-host microenvironment (9). The tumor-host microenvironment is composed of tumor cells, numerous types of host cells, extracellular matrices and various sources of secretory factors, which can modify the local extracellular matrix (ECM), stimulate migration and promote proliferation and survival (10). Proteases are fundamental to numerous biological processes and are associated with a wide variety of pathological conditions, including cancer (11). Matrix metalloproteinases (MMPs) are a large family of calcium-dependent zinc-containing endopeptidases, which are responsible for tissue remodeling and degradation of the ECM (12). As digestion of the ECM is essential for tumor invasion and metastasis, the role of MMPs in the later stages of tumor development has been studied (13).

A disintegrin and metalloproteinases (ADAMs) are a family of proteins with a sequence that exhibits similarities to the reprolysin family of snake venom metalloproteinases (14); ADAMs share the metalloproteinase domain with the MMPs (15). Functional ADAMs are involved in ectodomain shedding of diverse growth factors, cytokines, receptors and adhesion molecules (16). Furthermore, pathologies, such as inflammation and cancer, involve specific ADAM family members, including ADAM10 (17). Dysregulation of ADAM10 in inflammation and disease has lead to the use of the catalytic domain of the protein as a therapeutic target; however, ADAM10 also appears to play important roles in normal states (18). *In vitro*, ADAM10 has been implicated in E-cadherin cleavage within keratinocytes and gastric cancer cell lines (19,20). Moreover, in the prostate, the membranous ADAM10 expression was observed to be high in benign prostatic hyperplasia patient samples, and the nuclear translocation of ADAM10 coupled with the androgen receptor was involved in human PCa tumor growth and progression (21).

Previous studies have indicated that ADAM10 participates in PCa development, however, the mechanism has not been investigated. In the present study, TNF-α was identified as a specific inducer of ADAM10 protein expression in the PCa cell line, PC-3, and demonstrated the regulatory function that exists between tumor necrosis factor (TNF)-α and ADAM10 regarding gene expression and protein levels. Furthermore, it was identified that TNF-α regulates ADAM10 through the p38 mitogen activated protein (MAPK)/necrosis factor (NF)-κB signaling pathway. In addition, the effects of ADAM10 on Fas ligand (FasL) and cell apoptosis were investigated.

## Materials and methods

### Reagents and antibodies

Recombinant human TNF-α, anti-TNF-α and anti-ADAM10 antibody were purchased from Sigma-Aldrich Chemie B.V. (Zwijndrecht, Netherlands). An RNeasy kit was provided by Gibco-BRL (Gaithersburg, MD, USA) and an enhanced chemiluminescence (ECL) kit was provided by Boehringer Ingelheim (Berlin, Germany). The following were obtained from Invitrogen Life Technologies (Carlsbad, CA, USA): Annexin V-fluorescein isothiocyanate (FITC) apoptosis detection kit, expression vector pGEX-4T-1, pcDNA™3.1/myc-His, fetal calf serum (FCS), RPMI-1640 medium, nitrocellulose membranes for western blot analysis, a polymerase chain reaction (PCR) kit and Lipofectamine™ 2000. FITC-labeled goat anti-mouse IgG (H+L), anti-p38MAPK, anti-phospho-p38MAPK and anti-NF-κB antibodies were obtained from Millipore (Billerica, MA, USA) and SB 203580 and pyrrolidine dithiocarbamate (PDTC) were purchased from Calbiochem (San Diego, CA, USA). The primers that were used in the present study were synthesized by Shanghai Sangon Company (Shanghai, China) and small interfering RNA (siRNA), small interfering ADAM10 (si-ADAM10) and non-silencing siRNA (si-control) were obtained from Santa Cruz Biotechnology, Inc. (Santa Cruz, CA, USA). Unless otherwise specified, all of the other reagents were of an analytical grade.

### Cell culture

The human PCa cells, PC-3 and Epstein-Barr virus-transformed B371 cells were obtained from the American Type Culture Collection (Manassas, VA, USA). The cells were maintained in RPMI-1640 medium, which was supplemented with 10% (v/v) heat-inactivated FCS and penicillin-streptomycin-mixed solution in a humidified incubator with 95% air and 5% CO_2_. The medium was changed every 2–3 days and the cell concentration was ~10^6^ cell/ml. The experiments were conducted on cells in exponential growth. The study was approved by the ethics committee of The People’s Liberation Army Mount Lu Sanatorium (Jiujiang, China).

### RNA preparation and PCR

Total RNA was isolated from the untreated control cells and the TNF-α-treated cells using the RNeasy kit according to the manufacturer’s instructions. PCR was performed according to the manufacturer’s instructions and the PCR products were resolved on a 100 g/l agarose gel and visualized using ethidium bromide transillumination under ultraviolet light. β-actin served as an internal control to evaluate the efficiency of cDNA synthesis and the PCR amplification. The primer sequences were as follows: ADAM10 forward, 5′-TCCACAGCCCATTCAGCAA-3′ and reverse, 5′-AGGCACTAGGAAGAACCAA-3′; and β-actin forward, 5′-TCACCCACACTGTGCCCATCTACGA-3′ and reverse, 5′-CAGCGGAACCGCTCATTGCCAATGG-3′

### Flow cytometric analyses of ADAM10 surface expression

Flow cytometry was employed to detect the ADAM10 protein expression on the surface of the cells. Following treatment with TNF-α, the nonspecific antibody-binding sites of the PC-3 cells were blocked via incubation with 5% rabbit serum in Dulbecco’s phosphate-buffered saline. Anti-ADAM10 antibody was subsequently added and incubated at 4°C for 30 min. After washing, the cells were incubated with FITC-labeled goat anti-mouse IgG and analyzed by flow cytometry (FACSCalibyr flow cytometer, BD Biosciences, San Jose, CA, USA).

### Western blot analysis

The samples were separated using 10% SDS-PAGE and transferred on to a nitrocellulose membrane. The western blot analyses were performed as previously described (22) with minor modifications. The blot was incubated using anti-p38MAPK, anti-phospho-p38MAPK and anti-NF-κB antibodies, and visualized with horseradish peroxidase-conjugated anti-rabbit IgG and an ECL-Plus chemiluminescence detection system (GE Healthcare, Pittsburgh, PA, USA).

### DNA transfection

The segments of FasL and ADAM10 in the human PC-3 cells were amplified using PCR. The PCR-amplified ADAM10 and FasL segments were subcloned into pGEX-4T-1 and pcDNA3.1/myc-His, respectively and the B371 cells were seeded into a 24-well plate at a density of 10^5^ cells/well. B371 cells were transfected with an ADAM10 and/or FasL expression vector for 5 h in serum-free media using Lipofectamine 2000, according to the manufacturer’s instructions.

### Measurement of FasL using ELISA

The concentrations of soluble FasL (sFasL) were measured using ELISA (R&D Systems, Minneapolis, MN, USA) according to the manufacturer’s instructions. All samples were run in duplicate and the average was obtained.

### RNA interference

To knock down ADAM10 expression at the mRNA level, 5×10^5^ cells/well were seeded in complete medium in a 24-well plate. ADAM10 siRNA was transfected using Lipofectamine 2000 according to the manufacturer’s instructions.

### Statistical analysis

All data are expressed as the mean ± standard deviation. Data were compared by one-way analysis of variance and pairwise comparison procedures were conducted using Tukey’s test. P<0.05 was considered to indicate a statistically significant difference.

## Results

### TNF-α induces ADAM10 expression

**Figure 1:**
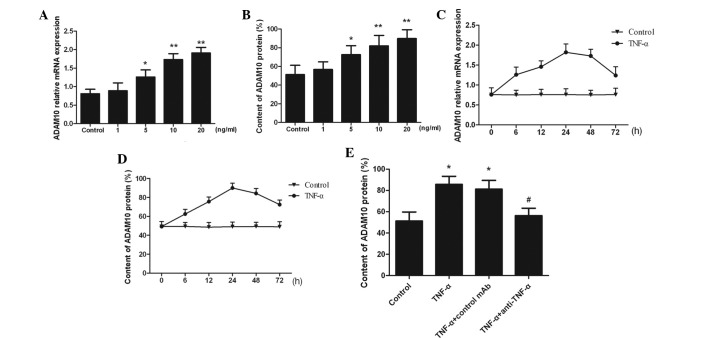
TNF-α induces ADAM10 expression in a time- and dose-dependent manner. PC-3 cells were treated with varying concentrations of TNF-α for 24 h and ADAM10 (A) mRNA and (B) protein expression was analyzed using PCR and flow cytometry, respectively. TNF-α (10 ng/ml) was used to treat PC-3 cells at various time-points and ADAM10 (A) mRNA and (B) protein expression was analyzed using PCR and flow cytometry, respectively. (E) ADAM10 expression was inhibited following the application of anti-TNF-α antibody. ^*^P<0.05 and ^**^P<0.01, vs. control; ^#^P<0.05, vs. TNF-α treatment group. ADAM10, a disintegrin and metalloprotease 10; TNF, tumor necrosis factor; PCR, polymerase chain reaction.

ADAM10 expression was observed in untreated PC-3 cells, however, expression was significantly increased following the addition of TNF-α in a dose-dependent manner (P<0.05; Fig. 1A and B). The ADAM10 expression increased in a time-dependent manner and peaked at 24 h in response to 10 ng/ml TNF-α stimulation (Fig. 1C and D). Moreover, the addition of anti-TNF-α neutralized TNF-α, and TNF-α-induced ADAM10 expression was attenuated (P<0.05; Fig. 1E). The results indicated that ADAM10 expression was markedly increased as a result of TNF-α stimulation in a time- and dose-dependent manner.

### TNF-α upregulates ADAM10 expression via the p38MAPK/NF-κB pathway

**Figure 2:**
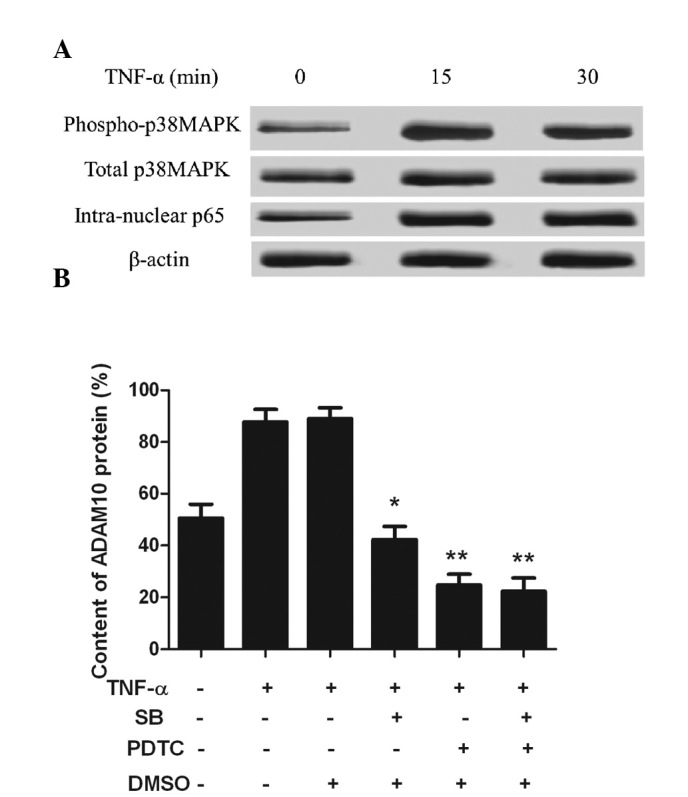
TNF-α upregulates ADAM10 expression via the p38MAPK/NF-κB pathway. (A) TNF-α (10 ng/ml) was administered to treat the PC-3 cells at three time-points. Western blot analysis showed that phosphorylated-p38MAPK and intranuclear-NF-κBp65 were markedly increased from 15 min. (B) PC-3 cells were pretreated with the p38MAPK inhibitor, SB, or the NF-κB inhibitor, PDTC, for 1 h and incubated with 10 ng/ml TNF-α for 24 h. Flow cytometry of the ADAM10 cell surface protein expression was conducted. ^*^P<0.05 and ^**^P<0.01, vs. TNF-α treatment group. TNF, tumor necrosis factor; MAPK, mitogen-activated protein kinase; ADAM10, a disintegrin and metalloprotease 10; NF-κB, nuclear factor-κB; SB, SB 203580; PDTC, pyrrolidine dithiocarbamate; DMSO, dimethyl sulfoxide.

As TNF-α-induced ADAM10 expression was upregulated via the p38MAPK/NF-κB pathway, phosphorylated-p38MAPK, p38MAPK and intranuclear NF-κB protein expression was measured in the PC-3 cells. Phosphorylated-p38MAPK and intranuclear NF-κB were rarely observed in the untreated control PC-3 cells, however, upon TNF-α stimulation for 15 min, the expression of the two significantly increased (Fig. 2A). The expression of ADAM10 was markedly reduced following addition of the p38MAPK inhibitor, SB 203580, and the NF-κB inhibitor, PDTC (P<0.05; Fig. 2B). These observations indicated that the ADAM10 expression was upregulated by TNF-α through the p38MAPK/NF-κB pathway.

### ADAM10 involvement in the cleavage of FasL

**Figure 3:**
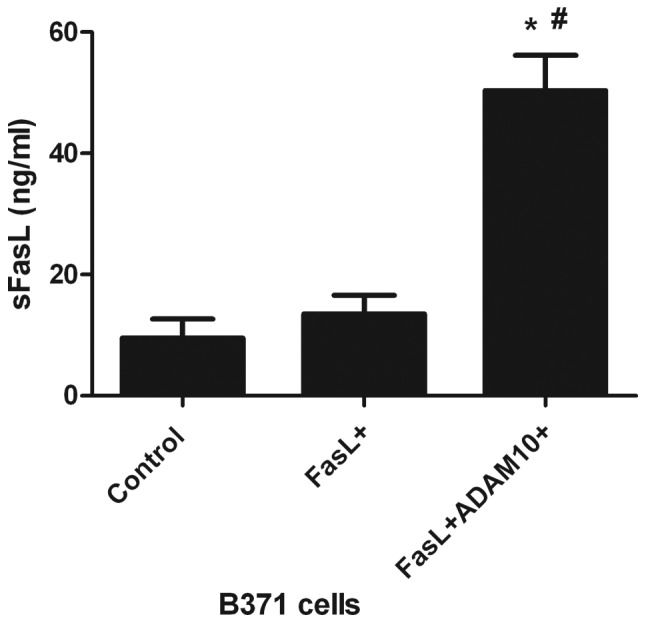
ADAM10 is involved in FasL shedding and results in increased sFasL release in the transfected B371 cells. B371 cells were transfected with recombinant FasL-6X His and/or recombinant ADAM10-glutathione *S*-transferase constructs for 24 h. Post-transfection, cells were harvested to measure the extent of apoptosis. ^*^P<0.05, vs. control; ^#^P<0.05 vs. FasL^+^B317 cells. sFasL, soluble Fas ligand; ADAM10, a disintegrin and metalloprotease 10.

To determine whether ADAM10 is responsible for FasL cleavage, the release of sFasL into the culture medium of FasL-transfected B371 cells (FasL-6X His) was investigated using ELISA. A marked effect on FasL shedding was observed in FasL^+^ADAM10^+^B371 cells, when compared with the B371 and FasL^+^B371 cells (P<0.05; Fig. 3). These data indicate that ADAM10 may be responsible for the cleavage of FasL.

### ADAM10 expression inhibits FasL-mediated apoptosis

**Figure 4:**
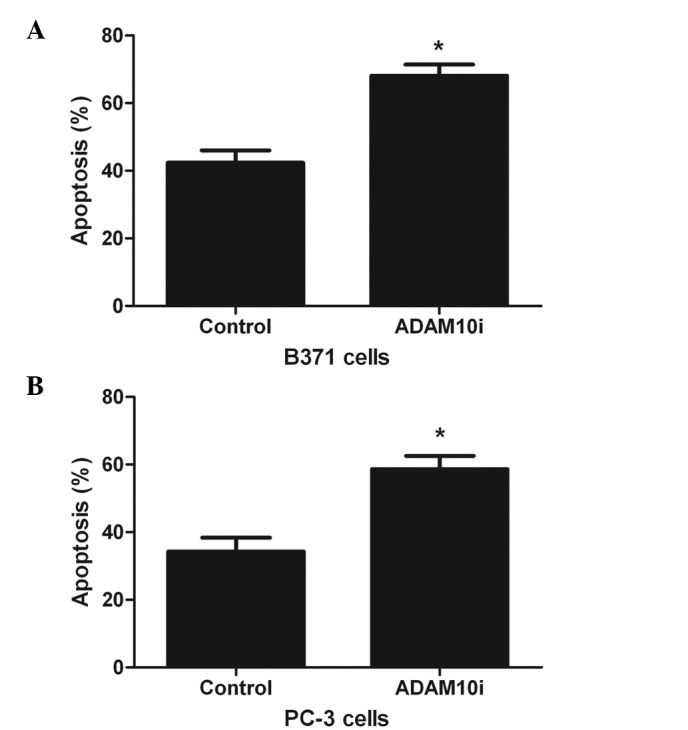
ADAM10 expression inhibits FasL-mediated apoptosis. B371 (FasL^+^ADAM10^+^) and PC-3 cells were transfected using control siRNA or ADAM10 siRNA for 24 h. Post-transfection, the cells were harvested to measure the extent of apoptosis. ^*^P<0.05, vs. control. ADAM10, a disintegrin and metalloprotease 10; siRNA, small interfering RNA; FasL, Fas ligand.

To further certify the involvement of ADAM10 in shedding, B371 (ADAM10^+^FasL^+^) and PC-3 cells were treated with ADAM10 siRNA and cell apoptosis was analyzed. The results indicated that compared with the control, treatment of B371 and PC-3 cells with ADAM10 siRNA resulted in a significant increase in apoptosis (P<0.05; Fig. 4A and B), therefore indicating that ADAM10 expression increases the resistance of the cells to apoptosis.

## Discussion

ADAM10 has been shown to exhibit substrate specificity, which overlaps with MMPs, thus indicating that ADAM10 has potential ECM-remodeling capabilities (23). Furthermore, ADAM10 may be critical during development and in adult tissues (21). ADAM10 is predominantly a sheddase, which is known to cleave epidermal growth factor (EGF)-like ligands from the cell surface and promote EGF receptor family signaling (24). TNF-α is a cytokine involved in systemic and acute inflammation (25). TNF-α has been implicated in inflammation-associated cancer and is produced by tumor cells and/or infiltrating leukocytes (26). In the present study, the results indicated that ADAM10 expression was increased following TNF-α stimulation in a time- and dose-dependent manner. Moreover, the TNF-α-induced ADAM10 expression was attenuated following the application of anti-TNF-α.

The p38MAPK signaling pathway is critical in normal immune and inflammatory responses (27) and is activated by numerous extracellular mediators of inflammation, including chemoattractants, cytokines, chemokines and bacterial lipopolysaccharides (28). TNF-α is able to activate the p38MAPK signaling pathway (29) and p38MAPK is key in the production of proinflammatory cytokines, in addition to being able to regulate cytokine expression by modulating transcription factors, such as NF-κB (30). Upon stimulation by TNF-α, TNF receptor (TNF-R)-1 recruits various groups of adaptor proteins, which are required for the activation of NF-κB inhibitor kinase (31) and induces the activation of the NF-κB signaling pathway (32). In the present study, TNF-α-induced ADAM10 expression, which demonstrated that in the local tumor microenvironment a variety of cells regulate the expression of tumor-associated molecules through paracrine and autocrine pathways, with important implications in the occurrence of tumors and their development and metastasis. p38MAPK and NF-κB inhibition resulted in lower ADAM10 expression, which indicate that TNF-α upregulated ADAM10 expression through the p38MAPK/NF-κB pathway in patients with PCa.

The cell surface-bound receptor Fas (also termed APO-1 or CD95) belongs to a subgroup of the TNF-R family, which contains an intracellular death domain and triggers apoptosis. Furthermore, FasL is a member of the TNF cytokine family (33). A previous study identified that cytotoxic T cells, which express FasL in its membrane-bound form (mFasL) on their surface, are able to kill Fas^+^ target cells (34). The Fas/FasL system is significant in tumorigenesis and a previous investigation has indicated that the impairment of the Fas/FasL system in cancer cells may lead to apoptosis resistance and contribute to tumor progression (35). ADAM10 downregulates the Fas/FasL signaling pathway through Fas shedding (36), which results in increased sFasL. In the present study, the results indicated that ADAM10 was involved in FasL shedding and ADAM10 expression inhibited FasL-mediated apoptosis. ADAM10, as an active metalloprotease, influenced the tumor via numerous pathways and may have participated in apoptosis-related protein hydrolysis. Malignant tumor development may, therefore, be associated with FasL loss by escaping the Fas/FasL scavenge system. sFasL is important during cell apoptosis as it is able to bind to Fas and act as an antagonist within the Fas/mFasL conjugate, which suppresses cell apoptosis. In PCa, increased levels of sFasL may compete with mFasL and bind to Fas, thus, blocking FasL-mediated apoptosis. Therefore, increased sFasL expression in the tumor cell microenvironment may be a mechanism of immune evasion. In conclusion, the present study indicated that ADAM10 hydrolyzed mFasL in patients with PCa, which increased the local sFasL concentration. Further investigation is required to establish whether ADAM10 may serve as a target for chemotherapeutic agents.
